# A Hypomethylating Variant of *MTHFR*, 677C>T, Blunts the Neural Response to Errors in Patients with Schizophrenia and Healthy Individuals

**DOI:** 10.1371/journal.pone.0025253

**Published:** 2011-09-28

**Authors:** Joshua L. Roffman, Adam Z. Nitenson, Yigal Agam, Marlisa Isom, Jesse S. Friedman, Kara A. Dyckman, David G. Brohawn, Jordan W. Smoller, Donald C. Goff, Dara S. Manoach

**Affiliations:** Department of Psychiatry, Massachusetts General Hospital and Harvard Medical School, Charlestown, Massachusetts, United States of America; University of Chicago, United States of America

## Abstract

**Background:**

Responding to errors is a critical first step in learning from mistakes, a process that is abnormal in schizophrenia. To gain insight into the neural and molecular mechanisms of error processing, we used functional MRI to examine effects of a genetic variant in methylenetetrahydrofolate reductase (*MTHFR* 677C>T, rs1801133) that increases risk for schizophrenia and that has been specifically associated with increased perseverative errors among patients. MTHFR is a key regulator of the intracellular one-carbon milieu, including DNA methylation, and each copy of the 677T allele reduces MTHFR activity by 35%.

**Methodology/Principal Findings:**

Using an antisaccade paradigm, we found that the 677T allele induces a dose-dependent blunting of dorsal anterior cingulate cortex (dACC) activation in response to errors, a pattern that was identical in healthy individuals and patients with schizophrenia. Further, the normal relationship between dACC activation and error rate was disrupted among carriers of the 677T allele.

**Conclusions/Significance:**

These findings implicate an epigenetic mechanism in the neural response to errors, and provide insight into normal cognitive variation through a schizophrenia risk gene.

## Introduction

Learning from mistakes is fundamental to adaptive, flexible behavior. To correct course, we must recognize and respond to errors, processes mediated by the dorsal anterior cingulate cortex (dACC). Individuals who mount stronger dACC responses to errors ultimately make fewer mistakes [1]. But what underlies variation in dACC function during errors? As suggested by previous work [2], [3], genetic factors likely contribute. However, epigenetic variables such as DNA methylation and chromatin remodeling could also modulate the brain's responsiveness to errors, as they play an essential role in neural plasticity [4]. While epigenetic phenomena cannot be measured directly in the living brain, we have used functional MRI to investigate whether a genetic variant in methylenetetrahydrofolate reductase (*MTHFR*) that strongly influences the one-carbon milieu also regulates dACC activity during error processing.

MTHFR is a key supplier of one-carbon moieties for intracellular methylation reactions, including DNA methylation and homocysteine metabolism. Specifically, MTHFR irreversibly reduces 5,10-methylenetetrahydrofolate (5,10-MTHF), which is derived from dietary folate intake, to 5-methyltetrahyrdofolate (5-MTHF). In turn, 5-MTHF supplies one-carbon moieties for downstream methylation reactions, including those catalyzed by methionine synthetase, DNA methyltransferases, and other vital transmethylation reactions. A common, well-characterized variant in the *MTHFR* gene, rs1801133 (677C>T) causes an amino acid substitution (222Ala>Val), each copy of which confers a 35% reduction in MTHFR activity [5]. Accordingly, individuals who carry the 677T allele exhibit lower genomic DNA methylation, especially in the setting of low serum folate, which supplies the substrate for MTHFR [6]. Reduced global DNA methylation has also been observed in heterozygous and homozygous *Mthfr* knockout mice [7], with numerous consequences for neurodevelopment and behavior. *Mthfr* −/− mice exhibit pronounced deficits including developmental retardation, altered cerebellar cytoarchitecture, and reduced survival at 5 weeks of age [7], while heterozygotes appear more grossly normal but exhibit hyperlocomotion and impaired recognition memory [8].

*MTHFR* 677C>T genotype has also been studied in relation to a variety of neuropsychiatric illnesses [9], [10]. A meta-analysis of 20 case-control studies indicated that the 677T variant augments risk for schizophrenia [11], a disorder characterized by blunted responses to errors [1] and rigid, perseverative behavior. Further, the 677T allele increases perseverative errors in schizophrenia [12], and was associated with diminished error-related dACC activation in a preliminary study of 18 patients [13]. However, as patient studies are confounded by the effects of chronic illness, including co-morbidities and antipsychotic use, it is unclear whether *MTHFR* effects on error processing in schizophrenia represent an epiphenomenon or a core aspect of the illness. Another possibility, one with broader implications for cognitive neuroscience, is that *MTHFR* exerts a more fundamental effect on error processing that transcends diagnosis, a plausible notion given the importance of one-carbon metabolism to normal brain development and function [4]. Here, we addressed this possibility by examining effects of *MTHFR* genotype on error-related brain activation in a cohort of 25 healthy individuals and 31 demographically matched patients with schizophrenia.

Participants underwent functional MRI using a 3.0 T scanner equipped for echo planar imaging (Siemens Medical Systems, Erlangen, Germany). During scanning participants performed a variant of the antisaccade task, which requires a gaze *away* from a suddenly appearing visual stimulus. Errors occur when participants fail to inhibit the prepotent response of looking *towards* the stimulus. Schizophrenia patients consistently show a higher antisaccade error rate and lower error-related activation of the dACC than healthy individuals [1]. We initially focused on regions-of-interest in the bilateral dACC, comparing error-related activation, based on the contrast of erroneous versus correct antisaccades, in C homozygotes and T allele carriers (i.e., C/T and T/T genotypes combined).

The dACC is a structurally and functionally heterogeneous region. In addition to its contributions to error processing, it is also thought to exert ‘top-down’ control on other ocular motor regions during preparation for antisaccades [14]. To determine the specificity of the *MTHFR* effect on error processing, we also examined whether *MTHFR* influenced dACC activation related to the preparation and execution of antisaccades, a different aspect of dACC function.

## Methods

### Ethics

The study was approved by the Partners HealthCare Human Research Committee. All participants provided written informed consent.

### Participants

The study included 31 outpatients with chronic schizophrenia, recruited from an urban mental health center, and 25 demographically matched healthy subjects, who were recruited from the community by poster and website advertisements (**Table 1**). Patients and healthy participants were different from those included in a previous MRI investigation [1], [13] that used a different version of the antisaccade task. Participants were excluded if they had a history of substance abuse or dependence within the previous 6 months, a history of significant head injury, or neurologic illness. Healthy participants were screened to exclude a personal history of Axis I mental illness [15] or a family history of schizophrenia-spectrum disorder. Patient diagnoses were confirmed using the Structured Clinical Interview for DSM-IV-TR [16]. Patients had been maintained on stable doses of second generation antipsychotics for at least six weeks, with the exception of one patient taking prolixin, and three patients who were not taking any medications.

**Table 1:** Characteristics of study participants.

	SCHIZOPHRENIA PATIENTS		HEALTHY PARTICIPANTS		
*MTHFR* genotype	C/C (n = 14)	C/T (n = 13) plus T/T (n = 4)	C/C (n = 13)	C/T (n = 9) plus T/T (n = 3)	*P* *
**DEMOGRAPHICS**					
Age at fMRI	40±3	45±3	41±3	39±4	n/s
Sex	12 M, 2 F	12 M, 5 F	10 M, 3 F	10 M, 2 F	n/s
Race	9 European, 5 Other	14 European, 3 Other	12 European, 1 Other	9 European, 3 Other	n/s
Length of illness (yrs)	19±4	19±3	–	–	n/s
Edinburgh handedness	64±14	57±13	57±15	67±8	n/s
**CLINICAL**					
PANSS positive	15±5	14±4	–	–	n/s
PANSS negative	14±5	15±5	–	–	n/s
PANSS general	30±9	31±8	–	–	n/s
CPZ equivalents	435±94	465±78	–	–	n/s
**PERFORMANCE AND MOTION**					
Estimated verbal IQ	101±3	97±3	106±4	110±4	healthy>patient, p = .01
Antisaccade latency (ms)					
• correct trials	300±16	277±14	276±12	272±14	n/s
• error trials	202±13	189±10	185±10	183±15	n/s
Antisaccade % error	26±6	32±5	13±3	18±3	patient>healthy, p = .004
Post-error slowing (ms; positive = slower)**	16±7	−4±7	13±8	7±8	C/C>T, p = .08
Average motion (mm)	2.33±0.28	2.43±0.25	1.98±0.29	2.31±0.30	n/s

Statistical tests were performed using ANOVA or chi-square as appropriate, with alpha (2-tailed) = 0.05. Abbreviations: fMRI, functional magnetic resonance imaging; PANSS, positive and negative syndrome scale; CPZ, chlorpromazine.

*n/s, no significant main effects of genotype or diagnosis, or significant genotype×diagnosis interaction.

**Post-error slowing was calculated as the difference in saccadic latency between correct trials following an error and correct trials immediately prior to errors.

### MRI acquisition

Images were acquired with a 3.0 T Siemens Trio whole body high-speed imaging device equipped for echo planar imaging (Siemens Medical Systems, Erlangen, Germany) and a 12-channel head coil. The protocol included a high-resolution MP-RAGE structural scan. Functional images were acquired using a gradient echo T2* weighted sequence that included prospective acquisition correction (PACE) for head motion [17]. Thirty-two contiguous horizontal slices, parallel to the intercommissural plane, were collected interleaved (TR/TE/flip = 2000 ms/30 ms/90°, voxel size 3.1×3.1×3.7 mm).

### Antisaccade task

**Figure 1:**
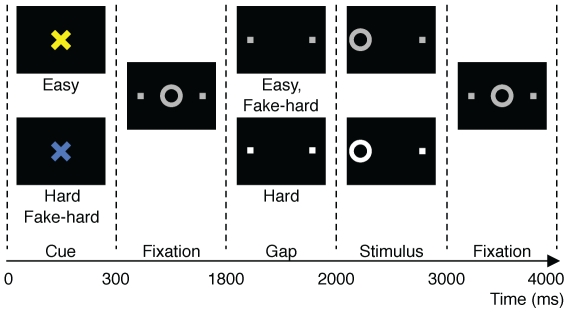
Schematic and timeline of the three trial types: Easy, Hard, and Fake Hard. For fMRI analyses comparing error and correct responses, we did not distinguish between trial types. All trials begin with an instructional cue (300 ms) of a color (blue or yellow) indicating either a Hard or Easy trial, followed by fixation. At 1800 ms, the central fixation ring disappears (200 ms gap), and at 2000 ms, it re-appears on either the right or left side as the imperative stimulus to which participants must respond. Hard trials are distinguished by an increase in luminance of both the peripheral squares that mark the potential locations of stimulus appearance during the gap and of the imperative stimulus. Except for the Hard cue, Fake Hard trials are identical to Easy trials. In the trials depicted, the correct response is a saccade *away* from the stimulus on the left side of the display. An error would involve a saccade towards the stimulus. After one second, the fixation ring returns to the center, where participants return their gaze to await the next trial.

We used a modified version of the antisaccade task described in [1] that consisted of three pseudorandomly intermixed types of antisaccade trials and fixation epochs (**Fig. 1**). Trials lasted 4 s and began with a central instructional cue for 300 ms, either a blue or yellow “X”, indicating whether the trial was “Hard” or “Easy.” Color assignment was counterbalanced across participants. The cue was followed by a white central fixation ring (1500 ms), and then a gap (200 ms) between the disappearance of the fixation ring and the appearance of the imperative stimulus, which appeared 10° to the right or left of the center. Participants were instructed to look away from the stimulus. “Hard” trials (40%) involved a distraction during the gap: a 3 dB luminance increase of the two peripheral squares that marked the potential locations of stimulus appearance. During easy trials (50%), luminance was unchanged. “Fake Hard” trials (10%) presented a hard cue but were otherwise identical to easy trials in that they lacked the luminance change; these trials were included as a control condition that would allow examination of the effects of a “Hard” versus “Easy” cue on fMRI activation unconfounded by the change in luminance that characterizes “Hard” trials. Participants practiced the task in a mock scanner. During fMRI scanning they performed six runs, each lasting 5 min 16 s, generating a total of 384 antisaccade trials and 120 fixation epochs. They received 5 cents for each correct response in addition to a base rate of pay. The ISCAN fMRI Remote Eye Tracking Laboratory (ISCAN, Burlington, MA) recorded eye position during scanning [see [1]]. Errors were combined across all trial types (“Hard,” “Easy,” and “Fake Hard”) for analysis.

### MTHFR genotype

DNA was extracted from whole blood samples, and MTHFR 677C>T genotype was determined using the MassArray platform (Sequenom, San Diego), using previously described primers [18]. As our a priori hypothesis focused exclusively on the MTHFR 677C>T polymorphism in relation to error-related dACC activation based on our preliminary study [13], this was the only genotype we investigated. Genotyping was conducted following acquisition of MRI scans. The observed minor allele (677T) frequency was similar (0.33) to that reported in the 1000 Genomes Project (0.32) [19].

### MRI analysis

Analyses were conducted on the cortical surface using FreeSurfer [20] and FS-FAST [21] software. Finite impulse response (FIR) estimates [21] of the event-related hemodynamic responses were calculated for error and correct trials for each participant. As in prior studies [1], [13], [22] we compared activation in error versus correct antisaccades at 6 s after trial onset, which is the peak of error-related dACC activation based on the averaged data of all participants. We also examined the contrast of correct antisaccades versus fixation at 4 s to determine whether *MTHFR* genotype affected activation related to generating a correct antisaccade. The correct antisaccades versus fixation contrast peaked at 4 s after trial onset across all participants.

Cortical activation was localized using an automated surface-based parcellation that delineated the cingulate cortex [23] and subdivided it into dorsal and rostral ACC, and posterior cingulate cortex regions [24]. Regions-of-interest (ROIs) were defined for the left and right dACC using both anatomical and functional constraints [see [13]; briefly, the dACC was defined using an automated parcellation of the cortical surface [25], and for each participant, the ROIs were functionally constrained to vertices within the dACC that showed error-related activation at p<.05 based on the averaged data of all 56 participants]. We averaged activation across all the vertices in each dACC ROI for each participant and used these values in repeated measures ANOVAs, with genotype and diagnosis as between-subject factors and condition (error or correct) and hemisphere as within-subject factors.

To explore effects outside of the dACC, we examined the effects of genotype (C/C versus T carrier) in the entire group (n = 56), as well as separately in patients (n = 31) and healthy participants (n = 25), on error-related activation across the entire cortical surface and subcortical brain volume. We also conducted a factorial genotype×diagnosis analysis for error-related activation. To correct for multiple comparisons, 10,000 Monte Carlo simulations of synthesized white Gaussian noise were run on the cortical surface and in the volume, using the same smoothing, resampling, and averaging parameters of the functional analyses. This determines the likelihood that a cluster of a certain size at a certain threshold (p<.01) would be found by chance (cluster-wise probability). These methods set the corrected overall probability level to 0.05 (2-tailed).

## Results

### Error-related dACC activation

**Figure 2:**
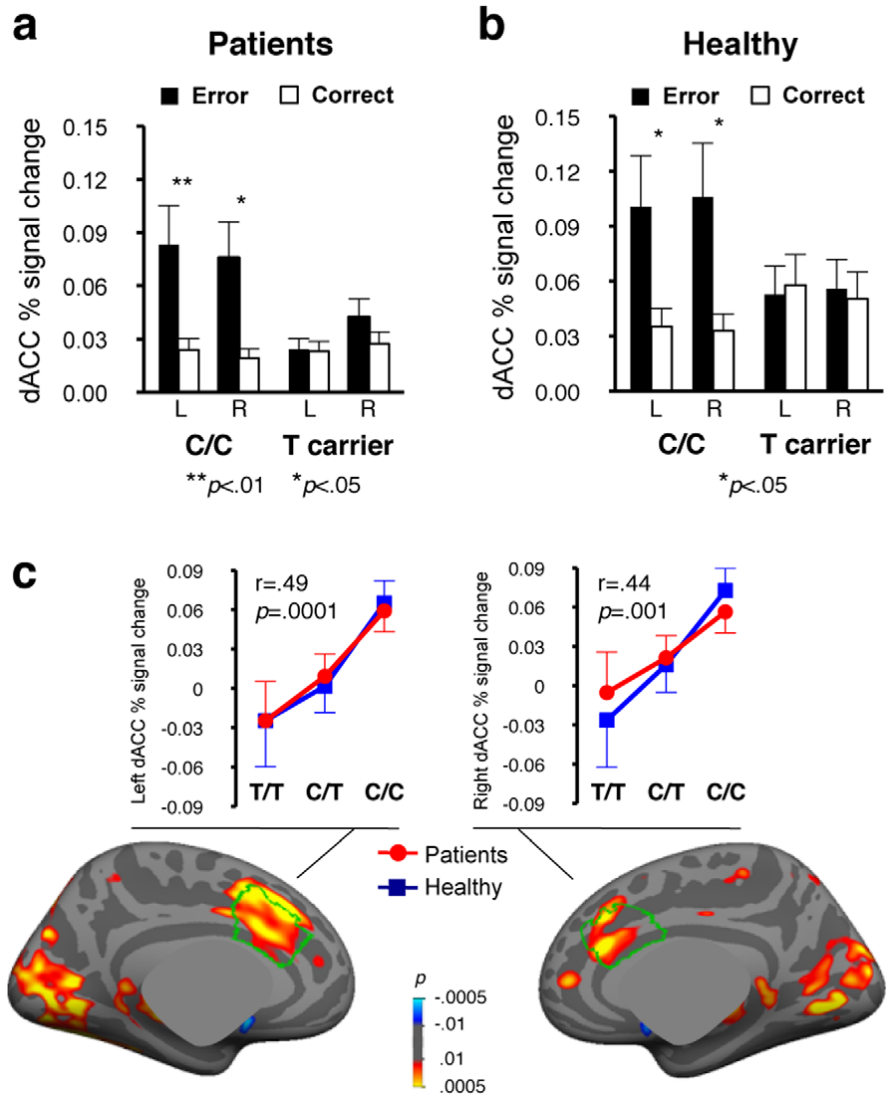
Effects of *MTHFR* 677C>T genotype on error-related dACC activation. Both schizophrenia patients (a) and healthy participants (b) exhibited significant condition×genotype interactions (patients: F = 4.51, p = .042; healthy participants: F = 10.32, p = .004) indicating that C/C participants, but not T allele carriers, showed significant error-related dACC activation. (c) Pseudocolor statistical maps of the relationship between 677C allele load (0, 1, or 2 copies) and error-related activation (error minus correct) in the combined group, displayed on the inflated medial cortical surface. The dACC is outlined in green. Graphs illustrate the effects of allele load on error-related activation, averaged across vertices in the anatomically defined dACC, for patients and healthy participants. Error bars indicate the standard error of the mean.

*MTHFR* genotype significantly influenced dACC activation bilaterally in the combined group of patients and healthy participants (genotype×condition interaction, F_1,52_ = 13.4, p = .001), with T allele carriers exhibiting blunted error-related activation in each diagnostic group (**Fig. 2a, b**). We observed no significant diagnosis×condition (F_1,52_ = 0.01, p = .92) or genotype×diagnosis×condition (F_1,52_ = 0.35 p = .56) interactions. As a test of possible population stratification artifact, the analysis was repeated using only participants of European ancestry (n = 44). Again, we observed significantly greater error-related activation in C/C than T allele carrier participants (genotype×condition interaction, F_1,40_ = 10.9, p = .002), with no significant diagnosis×condition (F_1,40_ = 0.19, p = .67) or genotype×diagnosis×condition (F_1,40_ = 1.5, p = .24) interactions.

**Figure 3:**
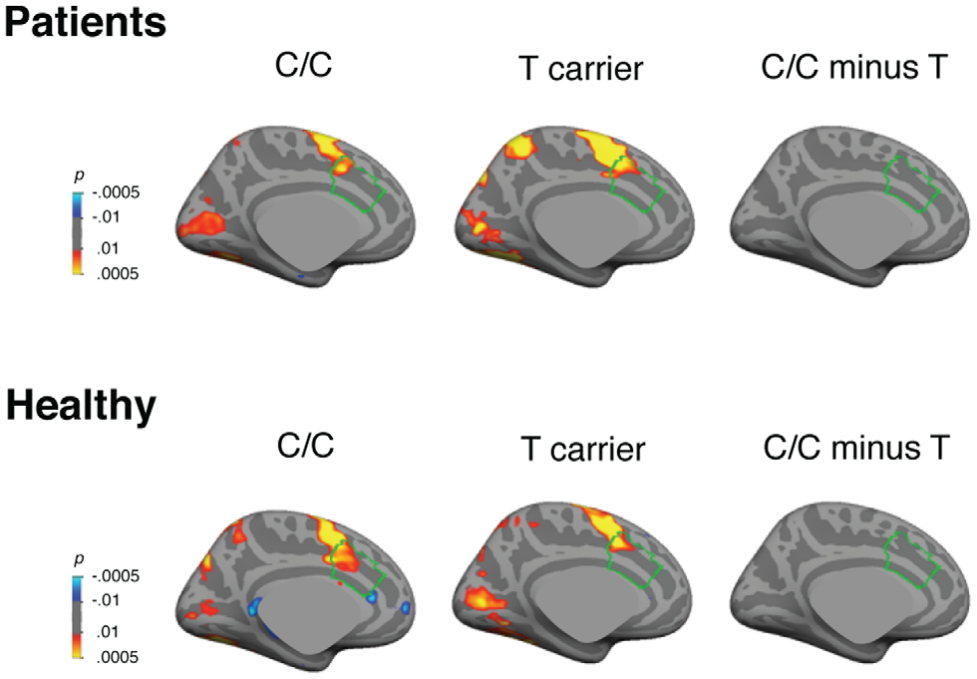
Pseudocolor statistical maps of MTHFR 677C>T genotype effects in the contrast of correct antisaccades versus fixation at 4 s displayed on the left inflated medial cortical surface. This contrast examines activation related to correctly performing an antisaccade at the time point that shows maximal activation in ocular motor regions in the combined group data. We observed robust activation in the ocular motor network, including in the cingulate eye field (which is within the dACC), that did not differ by genotype group. The dACC is outlined in green.

To determine the specificity of the *MTHFR* effect on error processing, we examined whether *MTHFR* influenced dACC activation related to the preparation and execution of antisaccades in the contrast of correct antisaccades versus fixation at 4 s [22]. Despite robust antisaccade-related activation of the dACC, there was no genotype effect (**Fig. 3**) suggesting that *MTHFR* effects were specific to error processing.

### Allele-load effects

As measures of intracellular methylation increase linearly with 677C allele load (0, 1, or 2 copies) [5], we next assessed whether a similar allele load effect would characterize error-related dACC activation. Linear regression in the combined group (n = 56) showed that error-related activation in bilateral dACC increased as a function of 677C allele load (left: r = .49, p = .0001; right: r = .44, p = .001), a pattern that was nearly identical in patients and healthy participants when examined separately (**Fig. 2c**). The results were similar when only participants of European origin were included in the analysis (n = 44; left: r = .51, p = .0004; right: r = .48, p = .001).

### Association with error rate

**Figure 4:**
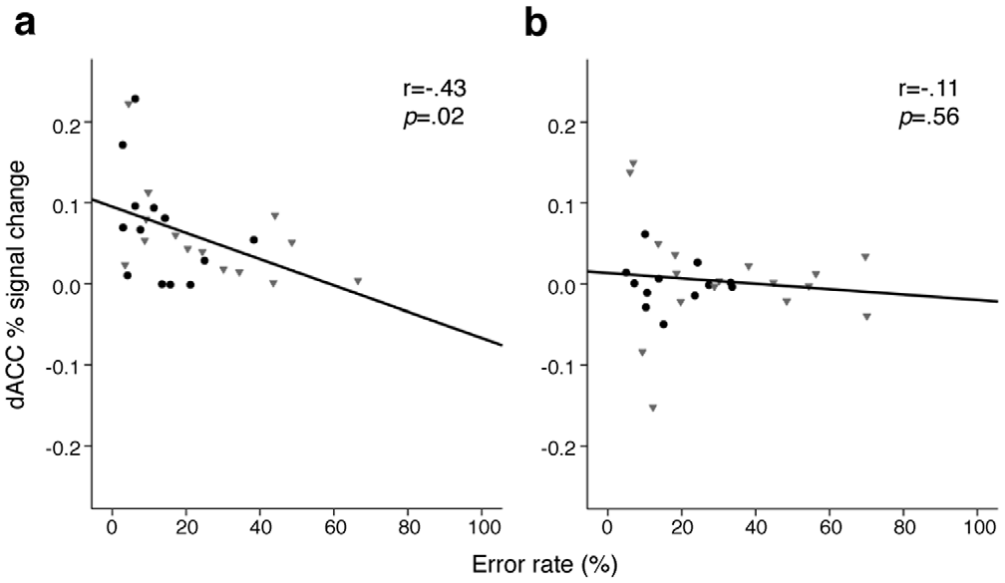
Relation of error-related dACC activation with antisaccade error rate in C/C (a) and T carrier (b) participants. Gray triangles indicate data points from schizophrenia patients; black circles indicate healthy participants.

Although 677T carriers made more errors, and showed a trend towards decreased post-error slowing, these effects did not reach significance (**Table 1**). This pattern is consistent with stronger effects of genotype on measures of brain function than performance, a common finding in imaging-genetics studies [26]. Only C/C participants, however, showed the expected [1] inverse relation of dACC activation with error rate (C/C r = .43, p = .02; T allele carrier: r = .11, p = .56; see **Fig. 4**), and this relation did not differ by diagnostic group. Among individuals of European origin, dACC activation again correlated with error rate among C/C (r = .44, p = .04) but not T allele carrier (r = .21, p = .33) participants.

### Whole brain analysis

To determine whether *MTHFR* genotype influenced error-related activation outside the dACC, we examined the effects of C/C versus T carrier genotype across the entire cortical surface and within the subcortical brain volume (**Table 2**). All analyses were controlled for multiple comparisons using cluster thresholds based on Monte Carlo simulations. Significant C/C>T effects on the inferior frontal gyrus were observed, regardless of diagnosis. Several additional regions that have previously been implicated in error processing [1], most prominently the bilateral insula, showed C/C>T effects in healthy but not patient participants. Conversely, in the left frontal pole, patients but not healthy participants exhibited significant C/C>T error-related activation. However, none of these regions exhibited significant genotype×diagnosis effects in the factorial interaction analysis conducted with all participants.

**Table 2:** Regions with significant (p<.05) effects of genotype on antisaccade error-related activation following whole-brain correction.

Hemisphere	Region	Maximal activation	Talairach coordinates (x, y, z)	Size (mm^2^)	Cluster-wise probability	Comments
**COMBINED GROUP (N = 56)**						
Left	Dorsal ACC	1.8×10^−6^	−9, 26, 33	1161	1.0×10^−4^	C/C>T carrier
	Frontal pole	4.6×10^−6^	−22, 53, 14	1146	1.0×10^−4^	C/C>T carrier
	Fusiform gyrus	8.5×10^−5^	−29, −75, −4	1313	1.0×10^−4^	C/C>T carrier
	Inferior frontal gyrus	1.2×10^−4^	−40, 24, 6	925	8.0×10^−4^	C/C>T carrier
Right	Dorsal ACC	1.8×10^−4^	7, 30, 20	607	2.5×10^−2^	C/C>T carrier
**HEALTHY PARTICIPANTS ONLY (N = 25)**						
Left	Fusiform gyrus	2.2×10^−5^	−28, −73, −3	1984	1.0×10^−4^	C/C>T carrier
	Anterior insula	7.4×10^−5^	−31, 27, −8	1135	1.0×10^−4^	C/C>T carrier
	Dorsal ACC	5.5×10^−4^	−10, 24, 33	509	2.8×10^−2^	C/C>T carrier
	Parahippocampal gyrus	6.5×10^−4^	−21, −33, −8	534	2.2×10^−2^	C/C>T carrier
	Supramarginal gyrus	7.7×10^−4^	−50, −50, 22	903	1.0×10^−3^	C/C>T carrier
	Superior parietal cortex	9.5×10^−4^	−19, −72, 42	1217	1.0×10^−4^	C/C>T carrier
Right	Anterior insula	3.8×10^−5^	35, 9, −9	1083	2.0×10^−4^	C/C>T carrier
**SCHIZOPHRENIA PATIENTS ONLY (N = 31)**						
Left	Frontal pole	7.2×10^−7^	−24, 56, 17	8192	2.3×10^−2^	C/C>T carrier
**GENOTYPE×DIAGNOSIS INTERACTIONS (N = 56)**						
Left	Fusiform gyrus	1.6×10^−4^	−24, −72, 0	1646	1.0×10^−4^	Healthy: C/C>T carrier, Patients: T carrier>C/C
	Hippocampus	1.7×10^−4^	−22, −33, −13	8776	1.4×10^−2^	Healthy: C/C>T carrier, Patients: T carrier>C/C
Right	Superior parietal cortex	4.9×10^−4^	40, −73, 38	7496	3.9×10^−2^	Healthy: C/C>T carrier, Patients: T carrier>C/C

Abbreviation: ACC, anterior cingulate cortex.

Two regions outside of the error processing network, the left fusiform gyrus and left hippocampus/parahippocampal gyrus, exhibited significant C/C>T error-related activation in healthy participants but not patients, as also reflected in a significant genotype×diagnosis interaction. A third region outside of the error processing network, the right superior parietal cortex, did not show significant genotype effects in either patients or healthy participants when these groups were studied separately, but did demonstrate a significant genotype×diagnosis interaction. In all three regions with significant genotype×diagnosis interactions, patients showed a reverse pattern of T carrier>C/C effects, although these effects did not reach significance in the patient-only group analysis.

## Discussion

The identification of genetic risk variants that influence neuropsychiatric disease risk or alter the course of illness has provided new insights into the neural and molecular mechanisms that underlie normal human cognition [26]. The *MTHFR* 677C>T variant is one of only a small number of functional polymorphisms that have been consistently associated with schizophrenia risk [11], and specifically with cognitive impairment among schizophrenia patients [12], [27]. Here, we found that both healthy participants and schizophrenia patients who carried the low-methyl 677T variant exhibited blunted error-related dACC activation, and that the magnitude of dACC activation was predicted by allele load. Previous work demonstrated that when genotype is not taken into account, the degree of error-related dACC activation predicts performance (error rate), suggesting the importance of intact dACC function to learning from errors [22]. However, here we found this relationship to be disrupted among all participants who carried the 677T allele, regardless of diagnosis. To the extent that *MTHFR* 677C>T genotype indexes genomic methylation status *in vivo*, as seen in previous work [6], [7], this finding suggests a novel epigenetic mechanism for understanding the neural response to errors.

In addition to its association with reduced global DNA methylation [6], the 677T allele also induces hypomethylation within glioblastoma cells [28] and promoter hypomethylation within colon cancer genes [29]. Similarly, altered DNA or histone methylation in the setting of the low-functioning 677T allele could influence the expression of other genes salient to error processing, although the mechanism by which these genes might be selectively targeted remains unknown. Previous work suggests that the 677T allele potentiates the metabolism of dopamine [12], [30], which has been postulated to modulate error-related dACC activation via striatal projections [31], and strongly influences prefrontally-mediated executive function [32]. However, other mechanisms could contribute to *MTHFR* effects on error processing circuitry; for example the 677T allele also inhibits the metabolism of homocysteine [6], which is toxic to dopamine neurons in culture [33]. It is also important to note that other regions in which dopamine neurons are putatively involved in error processing, such as the striatum, did not show *MTHFR* effects on error-related fMRI activation in the present study. Additional work is needed to understand the apparent selectivity of *MTHFR* 677C>T effects on dACC activation during error processing despite the more global effect of this variant on intracellular methylation processes.

The only previous study to examine effects of *MTHFR* genotype on brain function in healthy individuals found no significant effects of the 677T allele on working memory load-dependent prefrontal or dACC activation [30]. Among schizophrenia patients who participated in the same study, the 677T allele was associated with reduced dorsolateral prefrontal, but not dACC activation. This is consistent with our present findings that *MTHFR* genotype did not mediate the dACC response during the preparation and execution of antisaccades in either the patient or healthy groups. The dACC is thought to play a role in top-down control of motor regions while performing cognitively demanding tasks. Taken together these findings suggest an *MTHFR* effect on dACC function during error processing, but not on cognitive control during other executive function tasks (working memory or antisaccade generation).

Although the 677T allele has been consistently associated with increased schizophrenia risk [11], the mechanisms underlying this association remain uncertain. In the present study *MTHFR* genotype effects within the dACC were similar in similar patients and healthy participants, suggesting that by itself, the 677T variant does not contribute to error processing deficits in schizophrenia any more so than it does in the general population. However, in the context of altered prefrontal physiology, functional consequences of 677T allele-related dACC blunting during errors may be exacerbated in schizophrenia. Genome-wide profiling of DNA methylation using postmortem prefrontal cortex tissue has indicated numerous sites with altered methylation in schizophrenia, including genes that regulate glutamate and GABA signaling [34]. The presence of the hypofunctional 677T allele could augment these differences across a number of metabolic pathways that contribute to schizophrenia, not only those salient to error processing.

It is also noteworthy that error-related *MTHFR* genotype effects outside of the dACC differed in patients and healthy participants. Significant genotype×diagnosis interactions were observed in the left fusiform gyrus, left hippocampus, and right superior parietal cortex. These findings were largely driven by C/C>T carrier effects that were unique to healthy participants, although interestingly patients exhibited non-significant T carrier>C/C effects within these regions. These interactions were not expected, as they occurred in regions that are not typically associated with error processing. Within the error processing network, only healthy participants demonstrated significant C/C>T carrier effects in bilateral insula, but did not differ significantly from patients in this regard (i.e., the genotype×diagnosis interaction was not significant). The interpretation of these findings is not straightforward, and they should be considered preliminary until replicated. Still, in light of equivalent task performance by genotype within each diagnostic group, these patterns could provide insights into differing adaptations to *MTHFR* effects in patients and healthy participants, both within and external to the error processing network.

Epigenetic mechanisms are dynamic and reversible, even in mature neurons [35]. Folate status, in particular, may influence the degree to which hypofunctional *MTHFR* variants influence downstream methylation events. Among healthy individuals homozygous for the 677T allele, genomic DNA methylation and homocysteine metabolism are substantially lower than for those with C/C genotype, differences that are even more pronounced in the setting of low serum folate levels [6]. Similarly, among T/T schizophrenia patients, those with low folate levels exhibit more severe negative symptoms (which are closely related to cognitive impairment), while those with high folate exhibit more favorable negative symptom scores (similar to C/C and C/T patients, for whom negative symptom scores do not depend on folate levels) [18]. Among schizophrenia patients who carry the 677T allele and who receive folate supplementation, improvement in negative symptoms scores correlates with increase in serum folate level [36]. It is possible that folate level might also modulate the neural response to errors in 677T allele carriers and that folate supplementation might augment it, suggesting a potential intervention for blunted learning from errors.

### Limitations

There are several limitations to the present study. As direct observation of *MTHFR* genotype effects on downstream methylation measures is not possible *in vivo*, we cannot directly ascribe the *MTHFR* effect on error-related neural activation to specific patterns of DNA or histone methylation. In addition, dietary or serum folate levels were not available from study participants, precluding the study of interactive folate×*MTHFR* genotype effects on dACC function. The number of participants in each genotype×diagnosis group was relatively small. However, that the results were nearly identical in two groups (healthy participants and patients) and also echo those seen in a previous, smaller study of schizophrenia patients that used a different version of the antisaccade paradigm[13], suggests a low likelihood of Type I error. Finally, although study participants were predominantly Caucasian, the use of a racially admixed cohort raises the possibility of population stratification artifact; however, *MTHFR* genotype groups did not differ with regard to racial composition, and *MTHFR* effects persisted when only participants of European origin were included in the analysis, diminishing this concern.

### Conclusion

In summary, the present results demonstrate that a genetic variant implicated in schizophrenia risk alters the neural response to errors. They also suggest the importance of epigenetic control over error processing circuitry, implicating a novel molecular mechanism for how we regulate and flexibly modify our behavior.
